# Factors associated with self medication practice among pregnant mothers attending antenatal care at governmental health centers in Bahir Dar city administration, Northwest Ethiopia, a cross sectional study

**DOI:** 10.11604/pamj.2015.20.276.4243

**Published:** 2015-03-20

**Authors:** Gedefaw Abeje, Chanie Admasie, Belaynew Wasie

**Affiliations:** 1 Bahir Dar University, College of Medicine and Health Sciences, Bahir Dar, Ethiopia; 2 Ethiopian Red Cross Society Essential drug Program, Bahir Dar, Ethiopian

**Keywords:** Self medication, antenatal care, Bahir Dar city, Ethiopia, pregnant, mothers, health centers

## Abstract

**Introduction:**

Studies in different parts of the world indicate that there is high level use of self medication among pregnant women. But there are no scientific evidences on it and factors associated with it in Bahir Dar city administration. The aim of this study was therefore to assess level of self medication and identify factors associated with it among pregnant women attending ANC service at governmental health centers in Bahir Dar city administration.

**Methods:**

Institution based cross-sectional study was conducted from June 20-July10, 2013. Data were collected using structured questionnaire and analyzed using SPSS version16.0. Back ward logistic regression model was used to assess level of association with self medication practice.

**Results:**

A total of 510 pregnant women were included in the study. Of these, 25.1% reported self-medication during the current pregnancy. Self medication during pregnancy was significantly associated with gravida (AOR= 2.1, 95% CI: 1.3-3.4), maternal illness on the date of interview (AOR= 4.8, 95% CI: 2.9-8.0) and location of health facility (AOR= 4.6; 95% CI: 2.9-7.4).

**Conclusion:**

A considerable proportion of pregnant women practiced self-medication during their pregnancy with modern medications or traditional herbs. Mothers who were multi garvida, who had maternal illness on the date of interview and who were attending antenatal care were more likely to practice self medication.

## Introduction

In developing countries people use herbal medicines routinely in self-care. Some herbal medicines are potent, and their safety is not as evident as people think [1]. Globally information on the use of self medication during pregnancy is scarce. The attitude of pregnant women towards complementary and alternative drugs (CADs) seems to be an appealing approach to guarantee the well-being of their unborn children. Nevertheless, CADs are not always subject to the same regulations as conventional medicines and there is often little data concerning purity, safety and teratogenicity of this kind of medicines [2]. A study done in Addis Ababa Ethiopia on drug use among pregnant women reported that 12.4% of pregnant women practiced self medication [3]. Since the majority of women in Ethiopia live with poor access to health education, modern health care facility and qualified health professionals, the situation of self medication could be worst. But, there is no any study conducted on self medication among pregnant women in Bahir Dar city administration. Therefore, this study was conducted to assess self medication practice and associated factors among pregnant women in Bahir Dar city administration.

## Methods

The study was conducted in Bahir Dar city administration, Northwestern part of Ethiopia. Based on the 2007 population and housing census, population of Bahir Dar city administration was projected to be 239,721 for the year 2012 [4]. Institution based Cross- sectional study was conducted from June 20 -July10, 2013 in government health centers. All pregnant women who came for ANC service in Bahir Dar city administration were the source populations. All pregnant women who came for ANC services to the selected health centers during the study period were study populations. Pregnant women at any gestational age who were following the ANC service at the selected health centers were included in the study. Pregnant women referred from health institutions outside Bahir Dar city administration were excluded from the study. Pregnant mothers who came more than once during the study period were interviewed only once. This study was part of a study conducted to assess drug use among pregnant mothers attending antenatal care at government health centers of Bahir Dar city administration. Therefore, sample size used for that study was also used for this study. The sample size used for that study was calculated for single population proportion formula [5] taking proportion of drug use during pregnancy 71.3% from a study in Addis Ababa [3], tolerable margin of error 5% and design effect of 1.5. Adding 10% non response rate, the final sample size was 518.

Multi stage sampling method was used to select the required pregnant mothers. The health centers in Bahir Dar city administration were listed and stratified as rural and urban. From these health centers, two from the rural and three from the urban (five health centers) were sampled using simple random sampling method. Proportional numbers of pregnant women were assigned to each health center based on the flow of pregnant women per day calculated taking one previous month ANC record. Systematic random sampling method was used to select the pregnant women in each health facility. Information about socio-demographic data, obstetric and medical history and self medication practice of the pregnant women was collected from participants by semi structured questionnaire and by review of antenatal follow up cards using data collection format. Data were entered in to Epi-info version 3.5.2 and analysis was done using SPSS version 16.0. Bivarate analyses were performed by checking Hosmer and Lemeshow test and variables with p < 0.2 were included in multivariable logistic regression. Multivariable logistic regression was used to analyze association between important variables and self medication during pregnancy using back ward logistic regression model. Ethical clearance was obtained from the ethics review committee of the Bahir Dar University. Data collection was conducted after explaining the purpose of the study to the participants and obtaining verbal consent.

## Results

### Socio demographic characteristics

The majority of the respondents (77.5%) were in the age group of 20 and 34 while 49 (9.6%) of them were 19 and below. The mean (+ SD) age was 26.5 (±6.0). Four hundred sixty eight (91.8%) respondents were married and 40(7.8%) were single. Regarding their educational status, 151(29.6%) were unable to read and write, while 37 (7.3%) of them were able to read and write only. One hundred eight (21.2%) completed primary education, 103(20.2%) had completed secondary school, while 111 (21.8%) had attended higher level education (Table 1).

**Table 1:** Household characteristics of study participants

Household characteristic	Cases(n)	%	Controls(n)	%	OR, 95% C.I.	p-value
Rural	33	57	68	56		
Urban	28	43	54	44	1.0(0.6-1.4)	0.88
**Distance from water source**						
0-30 minutes	59	97	120	98		
>30 minutes	2	3	2	2	0.5(0.1-3.6)	0.6
**No. of habitable rooms in house**						
One	41	67	85	70		
Two or more	20	33	37	30	0.9(0.5-1.7)	0.87
**Floor type**						
Wood, soil, other	53	87	107	88		
Cement or tiles	8	13	15	12	0.9(0.4-2.3)	0.87
**Lighting fuel**						
Firewood, Paraffin	58	95	118	97		
Electricity	3	5	4	3	0.7(0.1-3.0)	0.69
**Ownership of mosquito nets**						
None	4	7	7	6		
One or more	57	93	115	94	1.2(0.3-4.1)	1
**Access to media**						
**Radio**						
Yes	51	84	106	87		
No	10	16	16	13	0.8(0.3-1.8)	0.65
**Television**						
Yes	3	5	4	3		
No	58	95	118	97	1.5(0.3-7.0)	0.69
**Internet**						
Yes	18	30	25	20		
No	43	70	97	80	1.6(0.8-3.3)	0.2

### Obstetric and medical history

Sixty five (12.7%) of the pregnant women reported history of chronic disease. Majority of the pregnant women (54.3%) were primigravida and 233(45.7%) were multi-gravida. Three hundred sixty two (71%) of the pregnancies were wanted, 78(15.3%) were mistimed; and 70 (13.7%) were unwanted pregnancies. Forty three (8.4%) of the respondents were hospitalized during their pregnancy. Three hundred ninety nine (78.2%) of the respondents had 1- 2 total ANC visits, and 111 (21.8%) of them had 3 - 4 total ANC visits on the date of interview. Three hundred forty nine (68.5%) of the pregnant women visited the health institutions for routine ANC follow up, 146 (28.6%) of them visited because of illness, 21(4.1%) referred from other health institutions of Bahir Dar city administration, and 2 (0.4%) visited for other reasons (Table 2).

**Table 2:** Paternal socioeconomic characteristics of study participants

Paternal characteristic	Cases(n)	%	Controls(n)	%	OR, 95% C.I.	p-value
Income(Ksh)						
<3000	33	54	54	45		
3000 +	28	46	66	55	1.4(0.8-2.7)	0.27
Occupation						
None	2	3	1	1		
Peasant agriculture, Small business, other	58	96	119	99	4.1(0.4-46.6)	0.26
Highest education level attained						
Has not completed primary school	20	33	45	38		
Completed at-least primary school	40	67	75	62	1.2(0.6-2.3)	0.7
Religion						
Christian	59	98	114	95		
Other	1	2	6	5	3.1(0.4-26.4)	0.43
Marital status						
Monogamous	45	75	103	85		
Polygamous, Other	15	25	17	15	0.5(0.2-1.1)	0.1
Has received information on immunization						
Yes	60	100	117	98		
No	0	0	3	2	3.1(0.2-62.4)	0.51

### Self medication practice during pregnancy

Among, 356 pregnant women who reported history of illness during their pregnancy, 128(36%) reported history of self medication. From this, 88 (68.7%) reported use of modern medication while 27(21.1%) of the pregnant women took traditional preparations. The rest 13(10.2%) took both modern and traditional medication. Not all of the pregnant women with perceived illness took medication to their illness. Accordingly 228 respondents with perceived illness during their pregnancy did not take any medication for their illness. The reasons given for not using medication were fear of damage to the fetus 171(75%), minor illness (24.1%) others 9(3.9%) (waiting next appointment and considering as pregnancy symptom). Among those who took medication for their illness, the basis for self medication were because it is less costly 8(6.25%), assumed that their illness was minor 29(22.6%), long waiting time at the health facilities 15(11.7%), and because they used the drug before 62(48.4%). The source of drug for self medication were pharmacy/drug shop for 47(56.2%), left over drugs for 21(16.4%) friends/relatives for 6 (4.7%), self prescribed herbal preparations for 39 (30.5%) and others (market areas) for 3(2.3%) (Table 3).

**Table 3:** Maternal socioeconomic characteristics of study participants

Maternal characteristic	Cases(n)	%	Controls(n)	%	OR, 95% C.I.	p-value
**Income(Ksh)**						
<3000	47	78	91	75		
3000 +	13	22	31	25	1.2(0.6-2.6)	0.71
**Occupation**						
None	12	20	21	17		
Peasant agriculture, Small business, Other	48	80	101	84	1.2(0.5-2.6)	0.68
**Highest education level attained**						
Has not completed primary school	28	46	63	52		
Completed at-least primary school	33	54	59	48	0.8(0.4-1.5)	0.53
**Religion**						
Christian	59	98	119	97		
Other	1	2	3	3	1.5(0.2-14.6)	1
**Marital status**						
Monogamous	45	75	102	84		
Other	15	25	20	16	0.6(0.3-1.3)	0.23
**Has received information on immunization**						
Yes	59	97	120	98		
No	2	3	2	2	0.5(0.1-3.6)	0.6

### Factors associated with self medication practice during pregnancy

Multivariable logistic regression model revealed that only gravida, maternal illness on the date of interview and location of health centers were significantly associated (p < 0.05) with self medication practice during pregnancy. Multi gravida pregnant women were more likely to practice self medication (2.1 times) than those with primigravida (AOR= 2.1, 95% CI: 1.3-3.4). Similarly, pregnant women with maternal illness on the date of interview were more likely to practice self medication (4.8 times) than those who had no maternal illness on the date of interview (AOR= 4.8, 95% CI: 2.8-8.0). Pregnant women who were attending ANC service in the rural health centers were also more likely to practice self medication (4.6 times) than those who were in the urban health centers (AOR= 4.6, 95% CI: 2.9-7.4) (Table 4).

**Table 4:** Factors associated with self medication practice during pregnancy among pregnant women in Bahir Dar city administration Northwest Ethiopia June 20-July10, 2013

Variable	Self medication		COR,95% CI	AOR, 95% CI	P-value
	Yes	NO			
**Age of women**					
15-19	5	44	1	1	
20-34	97	298	2.9(1.1-7.4)	2.8(0.9-7.9)	0.056
35-42	26	40	5.7(2-16.3)	2.4(0.7-8.4)	0.159
Educational level of women					
No formal education	64	124	2.1(1.4-3.1)	1(0.6-1.8)	0.897
Attained formal education	64	258	1	1	
**Monthly income**					
100-800	42	116	1.3(0.8-2.1)	1.1(0.6-1.9)	0.872
801-1000	33	78	1.5(0.9-2.5)	0.9(0.5-1.7)	0.766
> 1000	53	188	1	1	
Gravida					
1	46	231	1	1	
> 1	82	151	2.7(1.8-4.1)	2.1(1.3-3.4)	0.002
Maternal illness					
No	24	223	1	1	
Yes	104	159	6.1(3.7-9.9)	4.8(2.9-8.0)	<0.001
Location of health centers					
Urban	64	319	1	1	
Rural	64	6	5.1(3.3-7.9)	4.6(2.9-7.4)	<0.001

## Discussion

One hundred twenty eight (36%) pregnant women who reported history of illness during their current pregnancy self medicated themselves. Among these 88(68.7%) took modern medicines and 27(21.1%) took traditional medicine. Thirteen (10.2%) of the respondents took both modern and traditional herbs for self medication. In this study, 25.1% pregnant women self medicated during their current pregnancy. Among these, 5.3% used herbal preparation for self medication. It is higher than a study done in Addis Ababa where the prevalence of self medication was 12.4% [3]. The reason for this high prevalence of self medication and herbal medication use may be that the current study is done in an area where health facilities are not as such adequate and accessible to pregnant women compared to that of Addis Ababa. Education level of the mothers is another reason. Hence, traditional medicine (TM) may be the only available source of health care within a reasonable distance [6]. However, these findings were comparable to a study done in Nigeria, Gharoro and Igbafe, which revealed that self medication practice was 26.8% [7]. The herbal preparations used by the pregnant women for self medication in the present study include Zingeber officinale (ginger), Allium sativum (garlic or nechi shinkurit), Zehneria scabra (aregresa), Hageenia abyyssinica (kosso), and Cucurbita pepo L (Duba). Ginger, garlic and aregresa were also reported to be used for self medication in Addis Ababa, Sweden and Norway [3, 8, 6]. Among the herbs mentioned used for self medication in this study, Zingeber officinale has abortficient, emmenagogue and mutagenic effects [9].

From 72 participants who obtained drug(s) from drug stores/pharmacies for self medication practice during pregnancy, 25 (4.9%) of them didn't inform their pregnancy to their provider and 55(10.8%) of the providers didn't ask them about their pregnancy. This result is similar with a study done in Addis Ababa [3]. Self medication practice was found significantly associated with location of health centers (high in rural areas as compared to urban areas). This could be due to poor awareness on risk of herbal drug use during pregnancy among pregnant women in rural areas, relatively low income and educational status among rural women, and health facilities are not adequate and accessible to pregnant women in the rural areas compared to urban women. The reasons pregnant women mentioned for self medication included cost, perceiving that their illness was minor, because they used the drug before and considered it safe and the health facilities were not functional (closed) at that specific time, This finding was comparable to other research findings [3, 6, 10].

## Conclusion

A considerable proportion of pregnant women self-medicated themselves with modern medications or traditional herbs. Self medication practices of the pregnant women during pregnancy were significantly higher among women who were multi gravida, who had maternal illness on the date of interview and among women who were receiving ANC in rural health centers.

**Recommendation:** Health education should be given on the risk of self medication during pregnancy to pregnant women. Further community based studies should be conducted to identify factors determining self medication practice among pregnant women since this institution based study cannot be generalized to the community.
